# Structural and functional analysis of a homotrimeric collagen peptide

**DOI:** 10.3389/fbioe.2025.1575341

**Published:** 2025-04-28

**Authors:** Xinling Zhang, Kexin Li, Nan Lu, Takafumi Takebayashi, Boyu Zhou, Hongbin Xie, Yufan Li, Xingyun Long, Xingjiong Qin, Hongyi Zhao, Jiying Dong

**Affiliations:** ^1^ Department of Plastic Surgery, Beijing Hospital, National Center of Gerontology, Beijing, China; ^2^ Institute of Geriatric Medicine, Chinese Academy of Medical Sciences, Beijing, China; ^3^ LivingPhoenix Regenerative Technologies Development (Chengdu) Co., Ltd., Chengdu, China; ^4^ Department of Plastic and Reconstructive Surgery, Graduate School of Medicine, The University of Tokyo, Tokyo, Japan; ^5^ SkinVision Lab, Shanghai Institute of Plastic Surgery and Aesthetic Medicine Industrial Innovation, Shanghai, China; ^6^ Department of Plastic Surgery, Peking University Third Hospital, Beijing, China; ^7^ Sichuan Academy of Social Sciences, Sichuan, China; ^8^ Department of Plastic and Reconstructive Surgery, Shanghai Ninth People’s Hospital, Shanghai Jiaotong University of Medicine, Shanghai, China

**Keywords:** collagen peptides, homotrimer, synthesis, heat stability, safety

## Abstract

**Objective:**

This study aimed to chemically synthesize a homotrimeric collagen peptide, evaluate its safety, and assess its effectiveness in promoting collagen synthesis.

**Methods:**

A homotrimeric collagen peptide was synthesized and structurally characterized using circular dichroism and infrared spectroscopy. Thermal stability was analyzed by TG-DSC, and molecular weight and amino acid composition were determined. *In vitro* cytotoxicity testing assessed safety, while UV-induced photoaging experiments evaluated its effects on collagen and elastin synthesis. *In vivo* studies in BALB/c mice examined its impact on collagen content, skin structure, and angiogenesis.

**Results:**

The synthesized collagen peptide exhibited high purity (99.1%) and an amino acid composition of glycine, proline, and hydroxyproline in a balanced ratio (15:17:13). Structural analysis confirmed a stable triple-helical conformation similar to type I collagen with excellent thermal stability (Tm = 326.15°C). Cytotoxicity testing showed no adverse effects on cell viability. *In vitro*, the peptide significantly enhanced collagen and elastin synthesis in fibroblasts. *In vivo*, intradermal and subcutaneous injection increased collagen content, improved skin structure, and enhanced microvessel density.

**Conclusion:**

This study presents a chemically synthesized homotrimeric collagen peptide with superior purity, structural stability, and biological efficacy in promoting collagen synthesis. Compared to previous studies, this biomimetic material exhibits exceptional thermal stability (Tm = 326.15°C) and a well-balanced amino acid composition, enabling applications in cosmetics and medical devices requiring heat sterilization (e.g., autoclaving), as validated by our patented method (China Patent No. ZL202410309842.9).

## 1 Introduction

Collagen, a key component of the extracellular matrix, supports intercellular signaling, cell adhesion, immune response, and tissue stability (Du et al., 2021). Collagen’s biocompatibility, biodegradability, and low immunogenicity make it widely used in wound repair, tissue engineering, and regenerative medicine (Du et al., 2021). However, natural collagen’s clinical use is limited by viral transmission, immunogenicity, low extraction efficiency, poor solubility, and gelation issues (Avila Rodríguez et al., 2018; Yang et al., 2015). Recombinant collagen, designed based on the amino acid sequence of natural collagen, has been developed using synthetic biology techniques, allowing for the production of collagen within engineered cells. Nevertheless, existing expression systems like efficient *Escherichia coli* and yeast lack the post-translational modifications found in animal cells, requiring the addition of corresponding recombinant enzymes (Bhatwa et al., 2021). Moreover, they exhibit poor thermal stability. Although collagen produced in plant and mammalian cell systems possesses complete triple helical structures and good thermal stability, their low yields make practical clinical applications challenging (Ramshaw et al., 2014; Werkmeister and Ramshaw, 2012).

Designed collagen peptides offer several advantages at the molecular level, including controlled structure, ease of chemical modification, low risk of viral transmission, and quantifiable production. These properties make them excellent biomimetic materials (Badylak et al., 2015; Gelse et al., 2003). Biomimetic materials derived from collagen peptides have found extensive applications in various fields, including urethral repair, skin substitutes, and facial soft tissue augmentation (Farndale et al., 2008). Collagen possesses a characteristic triple helical structure formed by the intertwining of three α-chains with the repetitive (Gly-X-Y) n sequence, creating a right-handed super helical structure of polyproline II-type peptide chains. In 2006, Kotch and Raines synthesized peptides with (Gly-X-Y) n collagen characteristic sequences using chemical methods, which could self-assemble into a triple helical structure (Sengupta and Heilshorn, 2010; Ramachandran and Kartha, 2006). The amino acid sequences of the three peptide chains of collagen can be completely or partially identical, leading to the classification of collagen peptides into homotrimeric and heterotrimeric forms. Designing homotrimeric collagen peptides is relatively simple as the three α-chains have only one arrangement during self-assembly (León-López et al., 2019). However, heterotrimeric collagen peptides, where the three peptide chains are not completely identical or do not align perfectly, present challenges due to difficulties in achieving a single composition and arrangement (Fallas et al., 2010). Notably, our chemically synthesized homotrimeric collagen peptide overcomes the thermal instability limitations of natural and recombinant collagens. Its triple-helical structure remains intact up to 326.15°C, a critical advancement for biomedical applications requiring high-temperature processing.

The design and study of biomimetic homotrimeric collagen peptides have a long history. Materials formed by self-assembly of collagen peptides offer strong operability and low immunogenicity, making them relatively safe biomimetic collagen materials (Wu et al., 2024). Various strategies such as π-π stacking, amphiphilicity, metal-ligand interactions, and electrostatic interactions have been established to drive the self-assembly of collagen peptides into collagen fibers (Svensson et al., 2013; Sun et al., 2019; He et al., 2016). Collagen peptide materials with different assembly morphologies have different applications. Kumar et al. reported that the collagen peptide KOD could form nano fibers with a triple helical structure through multi-level self-assembly, and these fibers wound together to form a hydrogel, which served as an effective biomimetic hemostatic coagulation material (Kumar et al., 2014). Pries et al. reported a metal-triggered self-assembling collagen peptide that can be used as a three-dimensional scaffold material for cell culture and cell encapsulation, exhibiting good biocompatibility (Pires et al., 2009).

In this study, we designed and synthesized a homotrimeric collagen peptide material with a stable triple helical structure and good thermal stability. We characterized its structure and thermal stability using circular dichroism and infrared spectroscopy, evaluated its safety through cytotoxicity testing, and conducted *in vitro* and *in vivo* experiments to clarify its ability to promote collagen synthesis. We hope that the design and study of this material can further promote the development of biomimetic collagen and address more clinical needs.

## 2 Materials and methods

### 2.1 Synthesis of homotrimeric collagen peptides

The synthesis followed EDC⋅HCl/HOBt condensation (Yang et al., 2014) using L-Proline (Pro), L-hydroxyproline (Hyp), and glycine (Gly).

### 2.2 Key steps

1) Monomer preparation:
  a) *Boc-Pro*: Pro (5 g) reacted with di-tert-butyl dicarbonate (Diboc, 10 mmol) in NaOH/BuOH (1:3 v/v) at 60°C for 2 h.
  b) *Hyp-OMe/Gly-OMe*: Hyp or Gly (5 g) esterified with SOCl2 (0.1 mol) in MeOH/EtOAc (1:1 v/v) under reflux for 4 h.
2) Tripeptide (GPH) synthesis:
  a) Boc-Pro (2 mmol), Hyp-OMe (2 mmol), and Gly-OMe (2 mmol) coupled sequentially using EDC•HCl (5 mmol) and HOBt (5 mmol) in THF/EtOAc (1:1 v/v).
  b) Reaction mixture stirred at 25°C for 24 h, filtered, and purified via liquid-liquid extraction (EtOAC/NaHCO3).
3) Polymerization:
  a) GPH (10 mg/mL) polymerized with WSC (10 mmol) and HOBt (10 mmol) in phosphate buffer (pH 7.4) at 37°C for 48 h.
  b) Product freeze-dried (LGJ-10FD, Beijing Song Yuan Huaxing) for storage.

### 2.3 Structural characterization

1) Purity and Amino Acid Analysis
  • LC-MS/MS: Samples (2.5 mg/mL in 1% phenol/HCl) hydrolyzed at 110°C for 24 h.
    ○ Column: Endeavorsil C18 (1.8 μm, 100 × 2.1 mm).
    ○Mobile phase: 0.1% formic acid-acetonitrile gradient (0.2 mL/min, 40°C).
• Purity calculation: (Total amino acid content/sample concentration) × 100%.
2) Molecular Weight Distribution
  • **GPC**: Agilent 1,260 system with aqueous eluent.
    ○Sample (5 mg/mL) analyzed using Origin software for polydispersity index (PDI).
3) Secondary Structure Analysis
  • Circular Dichroism (CD):
    ○Spectra recorded (190–260 nm, Chirascan PLUS) with quartz cuvettes pre-treated with 2 M HNO3.
    ○Data processed via Pro-Data Viewer (smoothing: 4 iterations) and CDNN for helix content.
4) Triple Helix Confirmation
  • **FTIR**: Perkin Elmer Spectrum Two with ATR crystal.
    ○Pe to bovine collagen (amide I: 1,650 cm^−1^, amide II: 1,550 cm^−1^).

#### 2.3.1 Immunogenicity assessment

Endotoxin levels were quantified using Pyrogent™ Gel Clot Assay (Beyotime Biotechnology) per ISO 10993–1.

1) Standard curve: 0–1.00 EU/mL prepared by diluting 1.0 EU/mL stock.
2) Sample preparation: Collagen peptides (5 mg/mL) dissolved in endotoxin-free water.
3) Detection:
  a) 10 μL sample +10 μL LAL reagent → 37°C incubation (20 min).
  b) Absorbance measured at 545 nm (BioTek Synergy H1) after chromogenic reaction.
4) Validation: Negative controls (PBS) and blank (endotoxin-free water) included.

### 2.4 In vitro and in vivo studies

#### 2.4.1 Cell culture

Fibroblasts were seeded in 96-well plates at an appropriate density of 8 × 10^3^ cells per well and cultured overnight at 37°C with 5% CO_2_.

#### 2.4.2 Cytotoxicity assay

Upon achieving a cell confluence of 40%–60%, cells were subjected to different treatments according to experimental group settings. The control group received 200 μL of cell culture medium, the positive control group received 200 μL of cell culture medium containing 10% DMSO, the blank control group received 200 μL of cell culture medium containing 10% PBS, and the sample groups received 200 μL of collagen peptide solution at varying concentrations (%V/V). After 24 h of incubation, cell viability was assessed using the MTT assay, and relative cell viability was calculated.

#### 2.4.3 Treatment

Fibroblasts were seeded in 6-well plates at a density of 3 × 10^5^ cells per well and incubated for 24 h. Subsequently, cells were treated with either the blank control group or different concentrations of collagen peptide samples for 24 h.

#### 2.4.4 RNA Extraction and qPCR analysis

Total RNA was extracted and cDNA synthesized using appropriate methods. The expression levels of β-actin, COL1A1, and COL3A1 genes were determined using qPCR, with β-actin as the internal reference, and the relative expression levels of target genes were calculated.

#### 2.4.5 Cell treatment and UVA irradiation

Fibroblasts were seeded in 24-well plates at a density of 8 × 10^4^ cells per well and treated according to experimental group design. After 24 h of treatment, the negative control group and sample groups were subjected to UVA irradiation (at a wavelength of approximately 340–380 nm, the exposure time was about 1,500 s). Cells were collected, RNA extracted, cDNA synthesized, and the expression levels of COL1A1, COL3A1 genes determined using qRT-PCR. Additionally, Elastin levels were assessed using ELISA.

#### 2.4.6 Mouse grouping and treatment

Six-week-old male BALB/c mice were divided into collagen peptide and control groups. BALB/c mice were chosen for their advantages in immunological studies, making them suitable for evaluating local inflammatory responses after collagen peptide injection. Additionally, their lighter skin color facilitates the observation of material morphology and adverse skin reactions. Collagen peptides (5 mg/mL) and physiological saline were injected into the dermis and subcutaneous shallow layer of the mice using a hydro-injection device. Tissue samples were collected at 1, 2, 3, and 4 weeks post-injection for subsequent analysis.

#### 2.4.7 Mouse skin and tissue section processing

After sample collection, the collagen content in the mouse skin dermis was analyzed using second harmonic microscopy. Skin samples from mice sacrificed at 1 and 4 weeks were subjected to Masson’s trichrome staining, COL1, COL3 immunohistochemistry staining, and HE staining to assess the effect of collagen peptides on skin structure. CD31 immunohistochemistry staining was performed to evaluate the effect of collagen peptides on neovascularization in mouse skin.

### 2.5 Statistical analysis

Quantitative data from the experiments were analyzed using descriptive statistics, including mean and standard error. Analysis of variance (ANOVA) and t-tests were performed using SPSS 22.0 statistical software to determine significant differences among different experimental groups. Graphs were generated using GraphPad Prism 8.0 to visually represent the experimental results. Error bars in the graphs represent standard errors, with p-values less than 0.05 considered statistically significant.

## 3 Results

### 3.1 Physicochemical properties determination of collagen peptide

The homotrimeric collagen peptide designed in this study is composed of repeated Pro-Hyp-Gly sequences, which are the most abundant tripeptide in natural collagen proteins and the most stable sequence in the triple helix structure (Persikov et al., 2005; Thiagarajan et al., 2008).

#### 3.1.1 Purity and amino acid content analysis of collagen peptide

The analysis by liquid chromatography-mass spectrometry (LC-MS) showed a total amino acid content of 3,686.52 mg/kg. The concentration of the sample after lyophilization was measured to be 3,720 mg/kg, indicating a purity of 99.1%. The main amino acid components of collagen peptide, glycine, proline, and hydroxyproline, are present in a ratio of approximately 15:17:13.

#### 3.1.2 Relative molecular weight and distribution of collagen peptide

**FIGURE 1:**
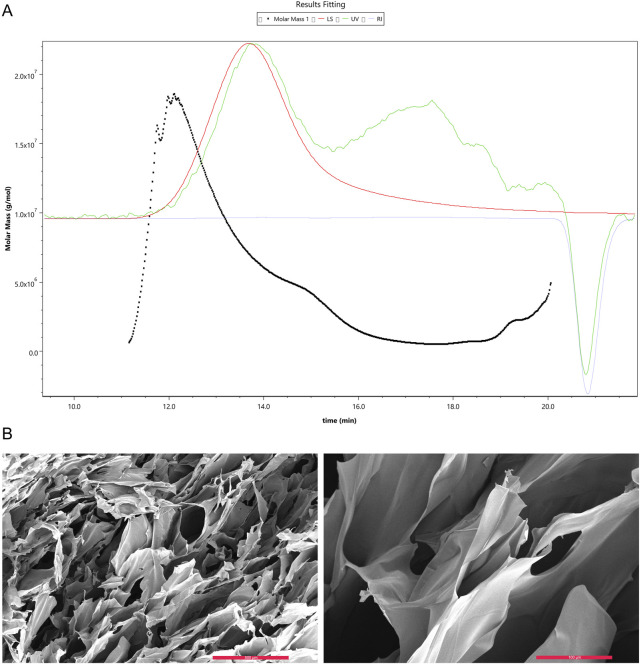
Gel Permeation Chromatography (GPC) analysis of collagen peptide molecular weight and scanning electron microscopy images. **(A)** Molecular weight of collagen peptide. Note: Mw stands for weight-average molecular weight, Mn for number-average molecular weight, Mp for peak molecular weight, and PD for polydispersity index. A higher value indicates a broader molecular weight distribution. **(B)** Scanning electron microscopy images of collagen peptide. Left: Displays the surface morphology of the collagen peptide, revealing its fibrous structure. The surface appears rough, with numerous pores and irregular features. Right: Provides a higher-magnification view of the collagen peptide, offering a more detailed look at its surface characteristics. The image highlights the intricate intertwining and interweaving of collagen fibers, forming a complex three-dimensional network.

The results of gel permeation chromatography (GPC) showed that the chemically synthesized collagen peptide has a molecular weight distribution ranging from 3.3 × 10^6^ to 1.8 × 10^8^ g/mol. The number of repeating units (*n*) was calculated using the formula n = (M_m_ - 18.01)/(285.13–18.01), corresponding to 12,354 to 673,854 repeat units. The polydispersity index (PDI) was determined to be 2.18 (Figure 1A). Observation of the self-assembled collagen peptide using scanning electron microscopy revealed a loose lamellar structure (Figure 1B).

### 3.2 Structural analysis of collagen peptide

#### 3.2.1 Secondary structure of collagen peptide with alpha helix, beta fold, turns, and irregular coils

Circular dichroism (CD) spectroscopy is a primary technique for analyzing protein secondary structures based on the differential absorption of left- and right-circularly polarized light by asymmetric molecules. By collecting circular dichroism (CD) absorption spectra of the collagen peptide and bovine type I collagen in the far-ultraviolet range (190–260 nm), and analyzing their secondary structures using software, it was found that the collagen peptide exhibits similar secondary structures to bovine type I collagen, including alpha helix, beta fold, turns, and irregular coils. Supplementary Table S2 showed the SCD Spectra Analysis.

#### 3.2.2 Stable triple helical structure of collagen peptide

**FIGURE 2:**
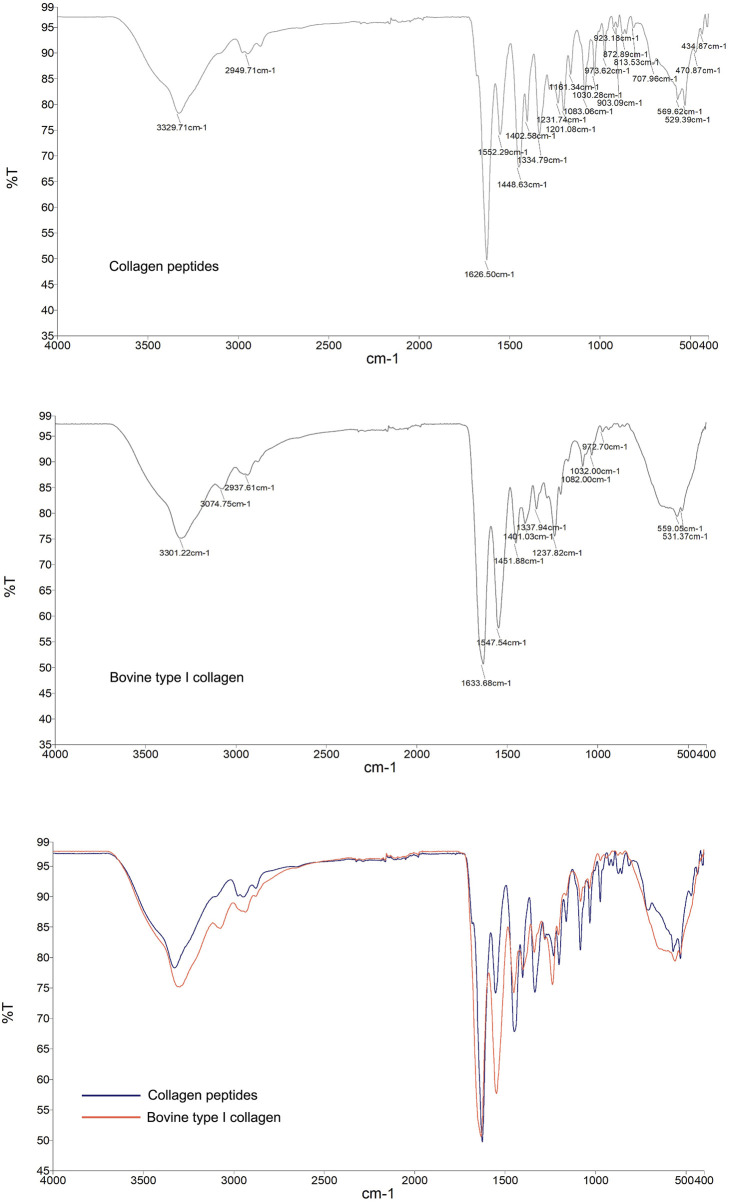
Infrared spectroscopy analysis of the molecular structural characteristics of collagen peptides. Reference to bovine type I collagen protein. Top Image: Infrared (IR) spectrum of the collagen peptide, showing its absorption peaks at different wavenumbers. Middle Image: Infrared (IR) spectrum of bovine type I collagen, displaying its absorption peaks at various wavenumbers. Bottom Image: Comparative infrared (IR) spectra of collagen peptide and bovine type I collagen. Blue curve: IR spectrum of the collagen peptide. Red curve, Red curve: IR spectrum of bovine type I collagen. The two samples exhibit similarities in the positions and intensities of their major absorption peaks, indicating that the collagen peptide shares molecular structural characteristics with bovine type I collagen.

Infrared spectroscopy, as a molecular vibration spectrum, is an important method for identifying the structure of organic compounds and the structure and conformation of biomacromolecules such as proteins. The collagen peptide, analyzed by protein infrared spectroscopy, exhibited characteristic peaks indicating the presence of a stable triple helical structure, similar to bovine type I collagen (Figure 2). Infrared Spectra Peak Characteristics were included in Supplementary Table S3.

#### 3.2.3 Collagen peptides exhibit excellent stability of triple helical structure

**FIGURE 3:**
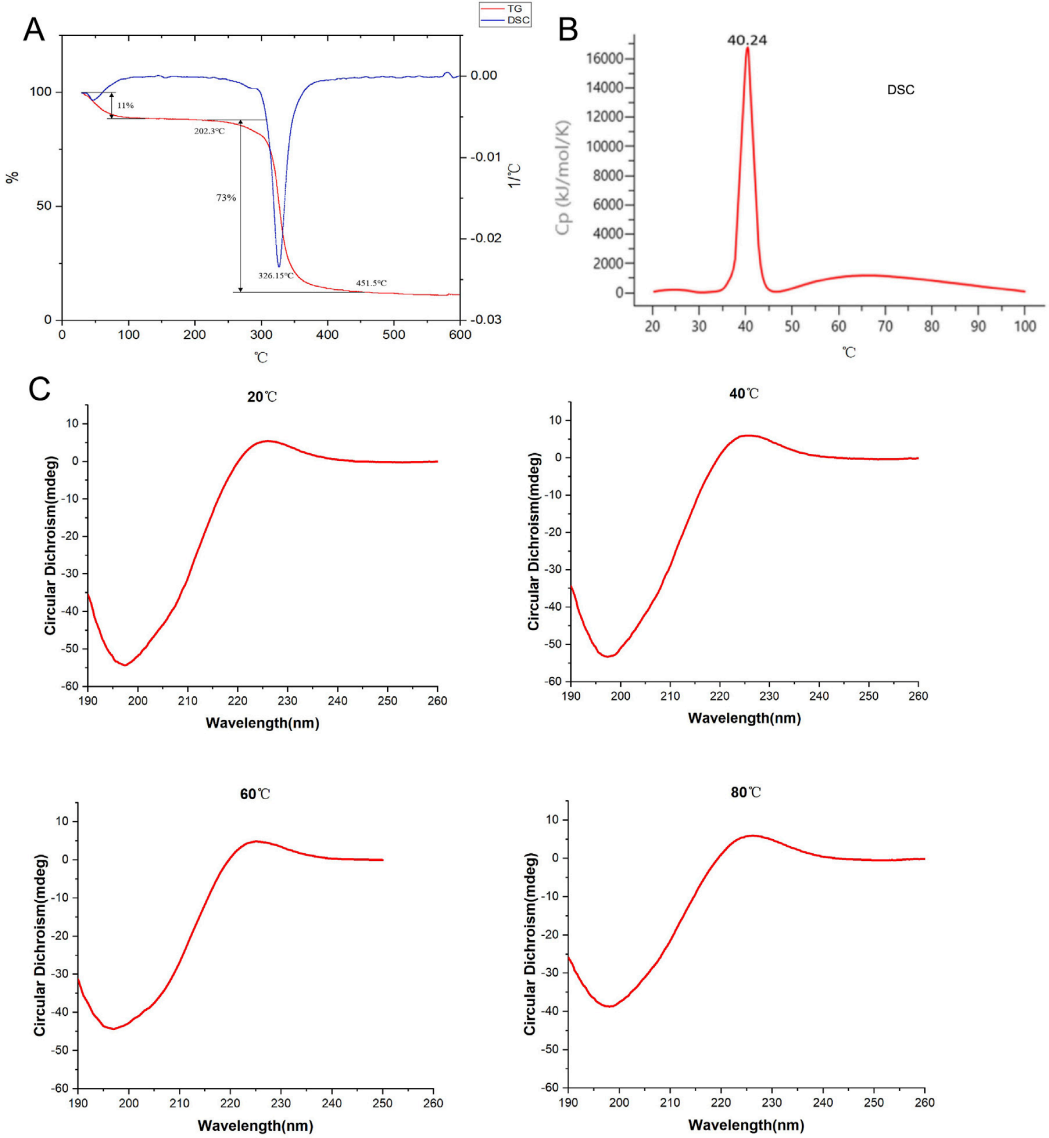
Detection of thermal stability of collagen peptides. **(A)** Analysis of the thermal stability of collagen peptides using TG and DSC methods. **(B)** Analysis of bovine type I collagen protein (as reference) using DSC method. **(C)** Assessment of the thermal stability of collagen peptides using circular dichroism spectroscopy at 20°C, 40°C, 60°C, and 80°C. Note: TG: Thermogravimetric Analysis; DSC: Differential Scanning Calorimetry.

The thermal denaturation temperature (Tm) reflects the temperature at which the triple helical structure of a protein unfolds into single chains, thus indicating the stability of the protein’s triple helical structure. Generally, higher Tm values indicate better thermal stability and greater stability of the triple helical structure. Differential scanning calorimetry (DSC) results showed that the Tm value of bovine type I collagen was 40.24°C, whereas the Tm value of collagen peptides was 326.15°C, significantly higher than that of bovine type I collagen. Additionally, simultaneous thermogravimetric (TG) analysis revealed that collagen peptides began to decompose at 202.3°C, reached almost complete decomposition at 451.5°C, with a maximum decomposition rate of 73%. Circular dichroism (CD) spectroscopy conducted during continuous heating showed that collagen peptides maintained a stable protein structure even at 80°C (Figure 3). These results indicate that collagen peptides possess excellent thermal stability and structural stability.

#### 3.2.4 Low immunogenicity of synthetic collagen peptides

To evaluate the immunogenicity of synthetic collagen peptides, endotoxin levels were quantified as a critical safety indicator.

• Standard curve validation: A linear relationship was observed between absorbance at 545 endotoxin concentrations (0.0625–1.00 EU/mL) (Supplementary Figure S1A).
• Sample analysis: All collagen peptide batches (n = 12) exhibited endotoxin levels ≤0.0625 EU/mL (Supplementary Figure S1B), significantly below the ISO 10993–1 threshold (0.5 EU/mL) for biomedical materials.
• Negative controls: PBS-treated samples showed no detectable endotoxin (0 EU/mL), confirming assay specificity.

These results demonstrate the low immunogenic potential of synthetic collagen peptides, addressing a key limitation of animal-derived collagens.

#### 3.2.5 *In vitro* promotion of collagen synthesis by collagen peptides

##### 3.2.5.1 *In vitro* cytotoxicity assay of collagen peptides

**FIGURE 4:**
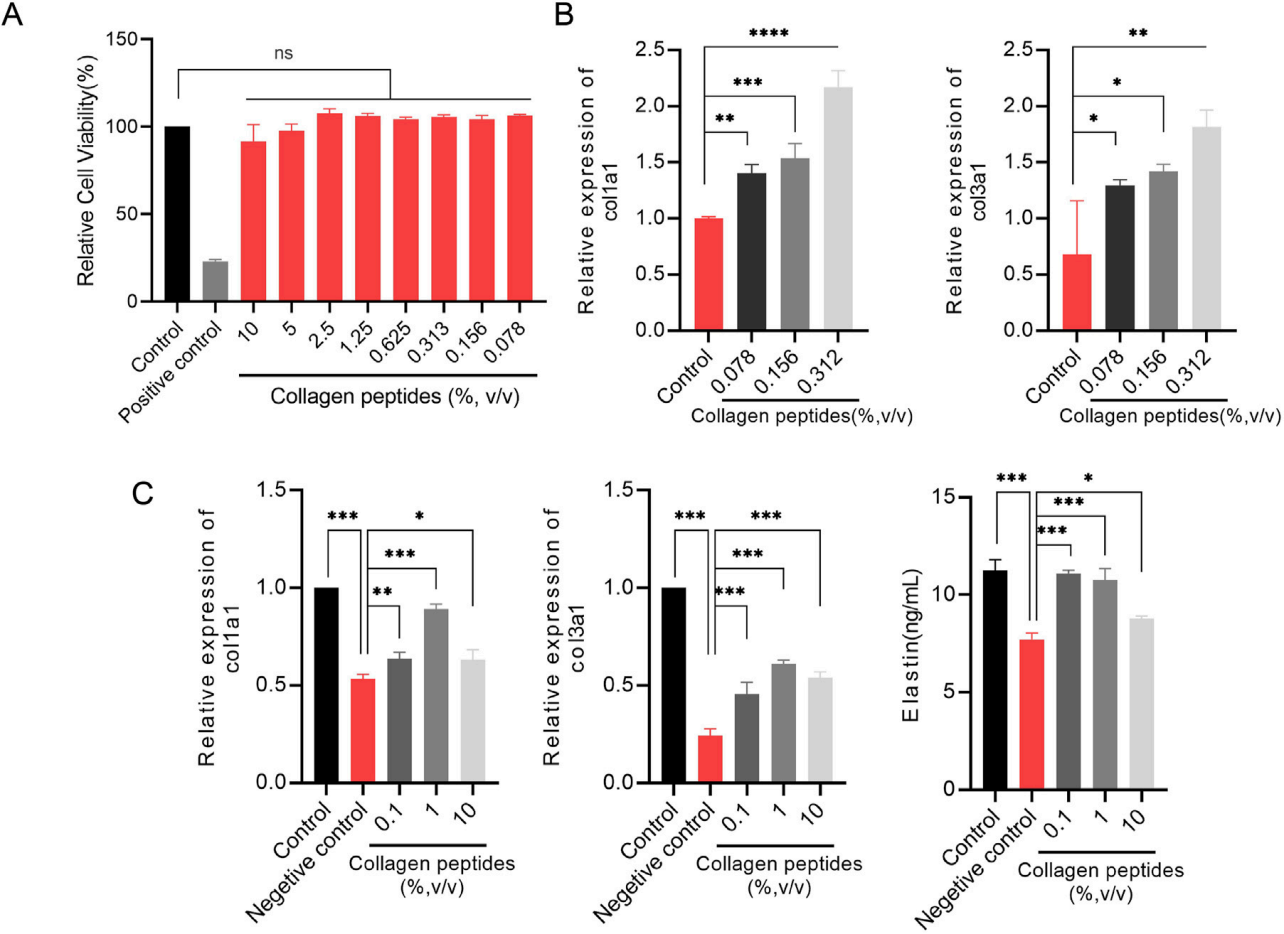
Collagen peptides promote collagen synthesis *in vitro* **(A)**. Cytotoxicity of collagen peptides at different concentrations. **(B)** The relative mRNA expression levels of type I collagen (col1a1) gene and type III collagen (col3a1) gene. **(C)** Collagen peptides can enhance the gene expression of col1a1, col3a1, and elastin in cells after ultraviolet radiation damage. Note: * indicates p-value <0.05; ** indicates p-value <0.01; *** indicates p-value <0.001.

To assess the cytotoxicity of collagen peptides *in vitro*, we used the MTT assay to evaluate the cell viability of fibroblasts treated with different concentrations (% v/v) of collagen peptides. The results showed that collagen peptides at concentrations up to 10% (v/v) did not exhibit cytotoxicity to fibroblasts compared to the blank control group (Figure 4A).

#### 3.2.6 Collagen peptides promote collagen synthesis in fibroblasts

To elucidate the *in vitro* promotion of collagen synthesis by collagen peptides in fibroblasts, we evaluated the relative expression levels of type I collagen (COL1A1) and type III collagen (COL3A1) genes by qPCR analysis. The results showed that fibroblasts treated with different concentrations (0.078%, 0.156%, 0.313%) of collagen peptides exhibited significantly higher relative expression levels of COL1A1 and COL3A1 genes compared to the control group, with a concentration-dependent effect. Higher concentrations of collagen peptides within a certain range could more effectively promote collagen synthesis in fibroblasts (Figure 4B). These results indicate that collagen peptides possess the ability to promote collagen synthesis in fibroblasts *in vitro*.

#### 3.2.7 Collagen peptides improve collagen and elastin synthesis capacities of fibroblasts after UV radiation exposure

UV radiation can decrease the expression of collagen and elastin in the skin, leading to skin laxity and wrinkles, among other signs of photoaging. We simulated the state of skin photoaging by subjecting fibroblasts to UV radiation (total dose of 9 J/cm^2^) *in vitro* and compared the expression levels of collagen type I (COL1A1) and type III (COL3A1) genes and elastin synthesis capacities of fibroblasts after supplementation with collagen peptides at concentrations of 0.1%, 1%, and 10%. qPCR results showed that UV radiation significantly decreased the gene expression levels of COL1A1 and COL3A1 in fibroblasts, while supplementation with 0.1%, 1%, and 10% collagen peptides significantly improved the expression levels of COL1A1 and COL3A1 genes after UV radiation exposure. ELISA results demonstrated that UV radiation significantly reduced the elastin content in fibroblasts, and supplementation with 0.1%, 1%, and 10% collagen peptides significantly improved the elastin synthesis capacity of fibroblasts after UV radiation exposure. Collagen peptides at appropriate concentrations could even partially offset the damaging effects of UV radiation (Figure 4C). These findings suggest that collagen peptides can improve skin laxity caused by photoaging by enhancing the synthesis of type I, type III collagen, and elastin.

### 3.3 *In vivo* injection of collagen peptides improves skin structure and promotes dermal proliferation in mice

**FIGURE 5:**
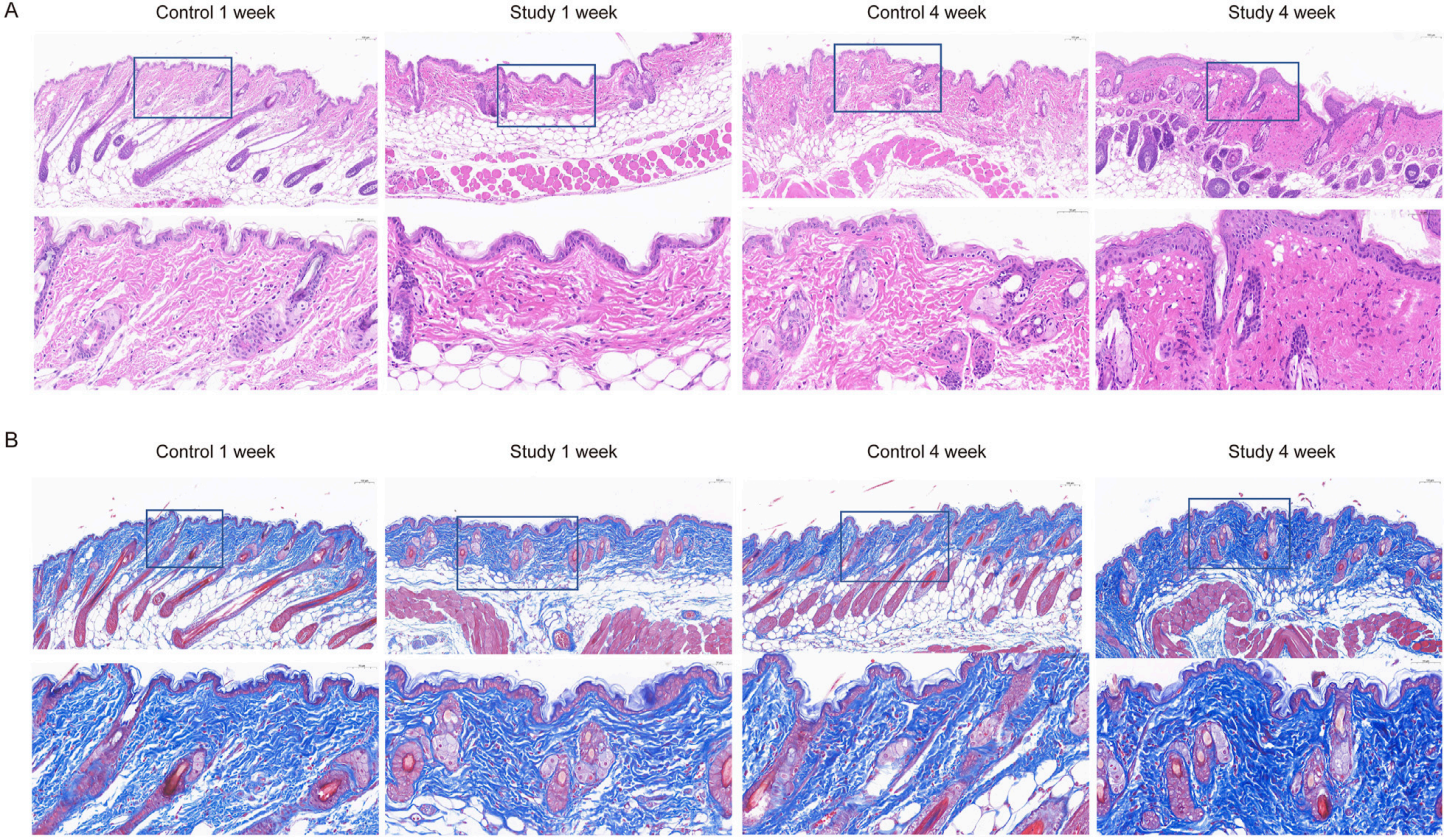
Collagen peptides can improve the structure of mouse skin and promote proliferation of the dermal layer. **(A)** HE-stained images of mouse skin tissue one and 4 weeks after collagen peptide injection. **(B)** Masson-stained images of mouse skin tissue one and 4 weeks after collagen peptide injection.

**FIGURE 6:**
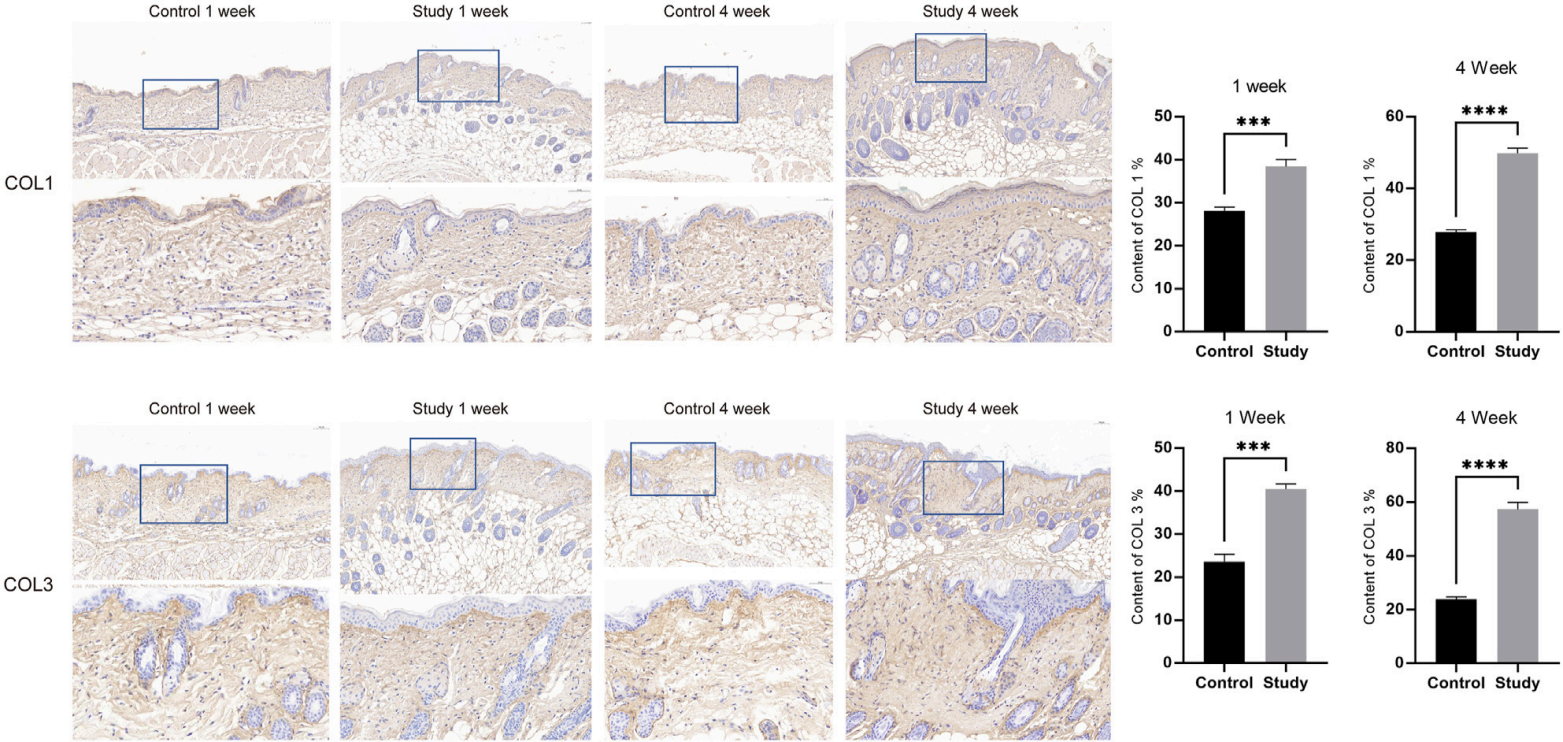
Injection of collagen peptides *in vivo* promotes collagen synthesis in mouse skin. Up image, COL1 expression in mouse skin tissue; Down image, COL3 expression in mouse skin tissue. Magnified views of specific regions showing detailed. Blue squares indicate areas of interest with higher magnification.

To explore the effects of *in vivo* injection of collagen peptides on mouse skin structure, we divided 6-week-old male BALB/c mice into experimental and control groups based on whether they were injected with collagen peptides or PBS, with 6 mice in each group and a total of 12 mice. Skin samples were collected and embedded in paraffin 1 week and 4 weeks after injection, followed by HE staining to observe changes in skin structure. The results demonstrated that injection of collagen peptides improved mouse skin structure and promoted dermal proliferation in a time-dependent manner. As time progressed, the proliferation of the dermal layer in the experimental group became more pronounced (Figure 5A). To further elucidate the effects of *in vivo* injection of collagen peptides on the collagen content in the dermal layer of mouse skin, we performed Masson’s staining, COL1 immunohistochemistry, and COL3 immunohistochemistry on skin samples collected at 1 week and 4 weeks after injection in both the experimental and control groups. The results showed that compared to the control group, *in vivo* injection of collagen peptides significantly increased the content of type I and type III collagen in the dermal layer of mouse skin, with a noticeable time-dependent effect. As time elapsed, the content of type I and type III collagen in the dermal layer of the experimental group mice increased significantly (Figures 5B, 6).

### 3.4 *In vivo* injection of collagen peptides promotes collagen generation in mouse dermal layer

**FIGURE 7:**
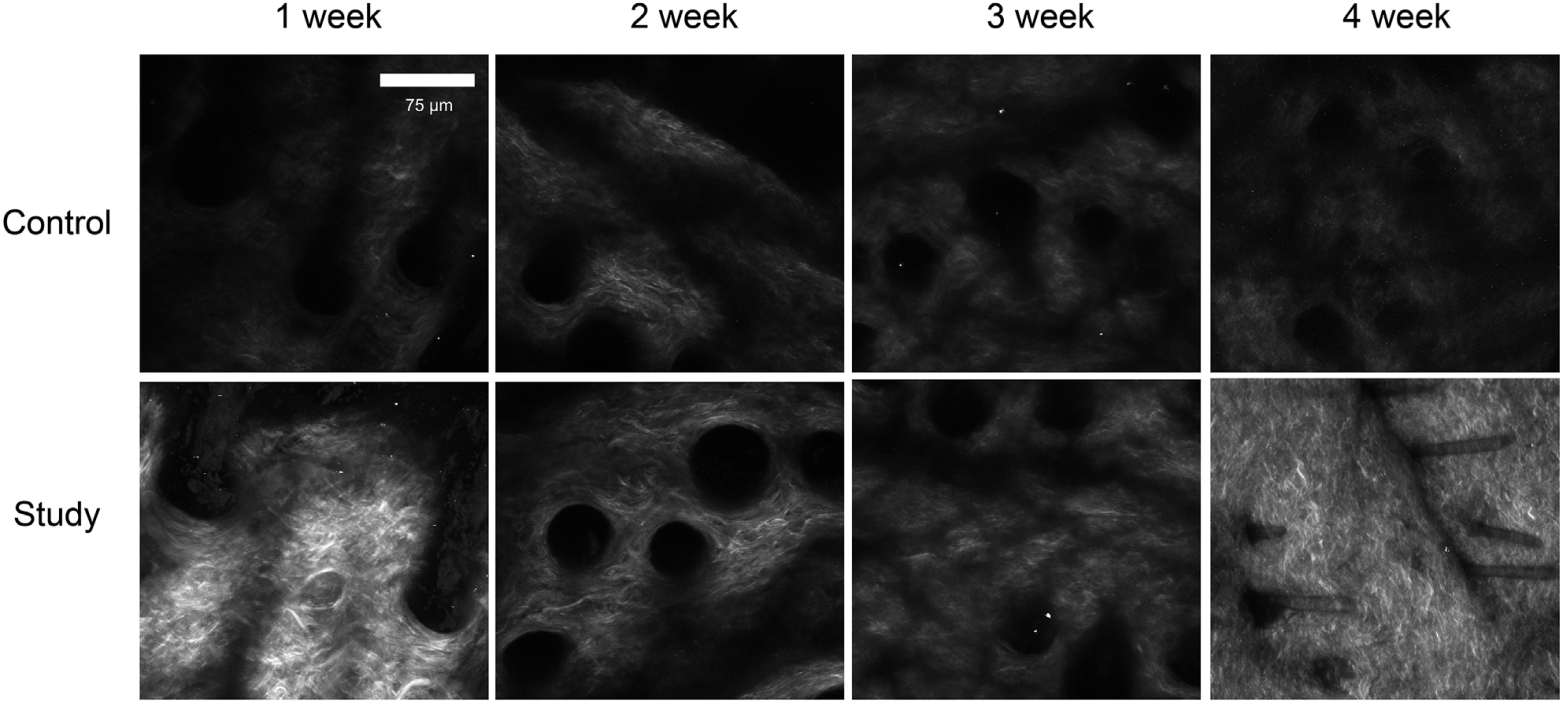
Observation of collagen in the dermal layer of mouse skin after collagen protein injection using second harmonic generation microscopy. Sparse collagen fibers with low density and limited network formation at all time points, with minimal structural changes over 4 weeks in control group. Increased collagen density and network formation at 1 week post-injection, with progressive enhancement in collagen fiber density and organization over time. By 4 weeks, a well-defined, dense collagen network was observed in study group.

To investigate the effects of *in vivo* injection of collagen peptides on collagen in the dermal layer of mouse skin, 6-week-old male BALB/c mice were divided into experimental and control groups based on whether they were injected with collagen peptides or PBS, with 12 mice in each group and a total of 24 mice. The mice were observed using second harmonic generation microscopy 1 week, 2 weeks, 3 weeks, and 4 weeks after injection to assess changes in collagen in the dermal layer. One week post-injection, the experimental group showed significantly higher dermal collagen content than the control group. However, at 2 weeks post-injection, while still higher than the control, the experimental group’s collagen content was significantly lower than at the 1-week time point. In weeks three and four after injection, the collagen content in the dermal layer of mice in the experimental group showed an increasing trend, with a clear time-dependent effect. We hypothesized that the initial increase in collagen content in the dermal layer of mice in the experimental group might be due to the residual collagen peptides in the skin, while the second increase might be attributed to the collagen peptides promoting the synthesis of new collagen in the dermal layer of the skin. To confirm this hypothesis, we directly detected collagen peptide samples using second harmonic generation microscopy and found a distinct second harmonic signal, thus partially confirming our hypothesis (Figure 7). These results indicate that *in vivo* injection of collagen peptides can provide initial support and filling effects, followed by the promotion of collagen synthesis in the dermal layer of mouse skin.

### 3.5 *In vivo* injection of collagen peptides promotes neoangiogenesis in mouse skin

**FIGURE 8:**
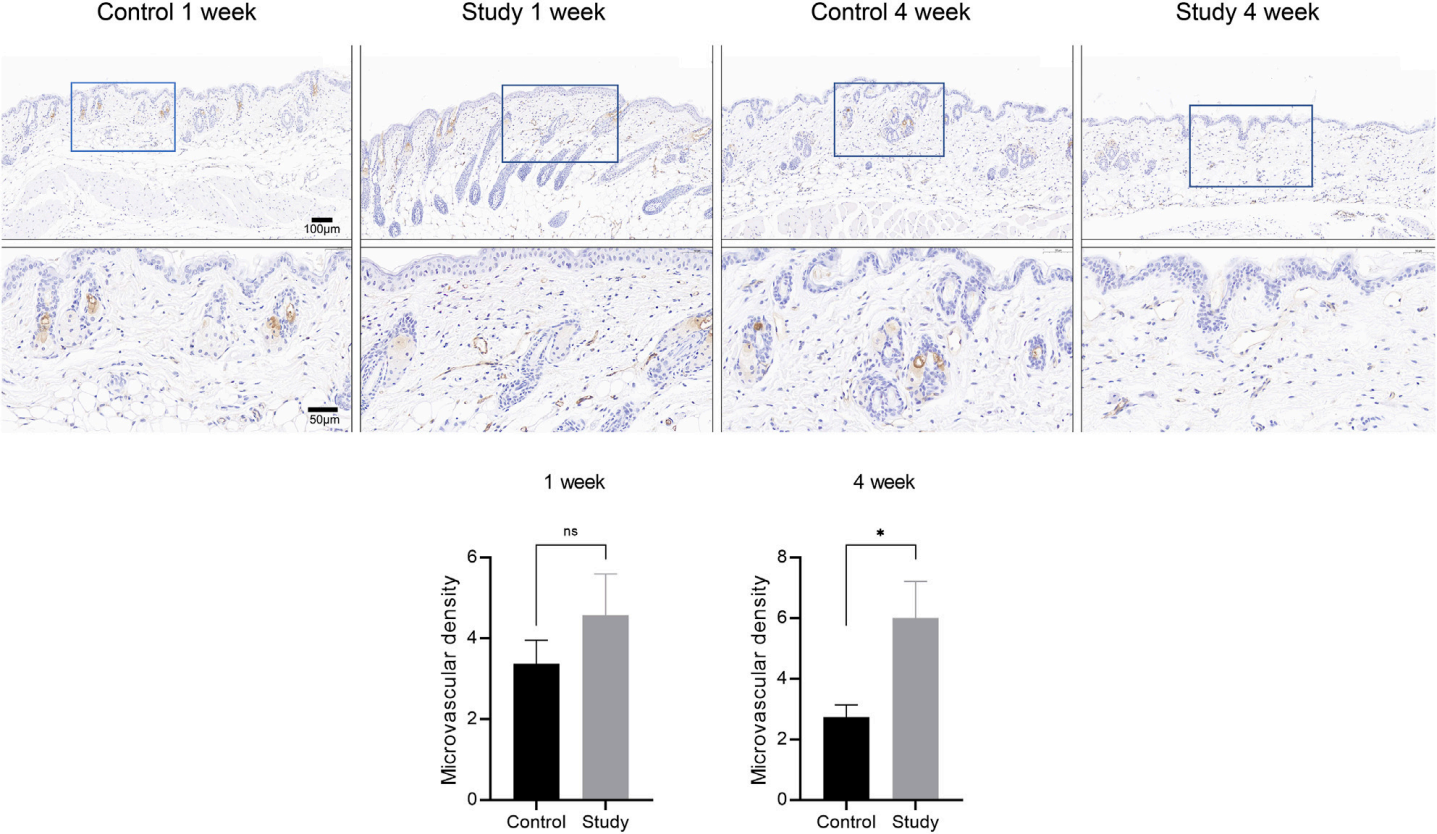
*In vivo* injection of collagen peptides promotes neovascularization in mouse skin. Immunohistochemical images of CD31 in skin tissue after one and 4 weeks of collagen peptide injection (up). Signal quantification graph in the immunohistochemical images of CD31 (down). Note: ns indicates p-value >0.05; * indicates p-value <0.05.

To explore the effects of *in vivo* injection of collagen peptides on neoangiogenesis in mouse skin, 6-week-old male BALB/c mice were divided into experimental and control groups based on whether they were injected with collagen peptides or PBS, with 6 mice in each group and a total of 12 mice. Skin samples were collected 1 week and 4 weeks after injection and subjected to CD31 immunohistochemical staining after paraffin embedding to observe the formation of new blood vessels in the skin. The results showed that *in vivo* injection of collagen peptides significantly promoted the formation of new blood vessels in mouse skin, with a noticeable time-dependent effect. As time progressed, the number of new blood vessels in the skin of mice in the experimental group increased significantly, and the vascular structure became clearer (Figure 8).

## 4 Discussion

Collagen, with its unique triple helical structure and biological functions, serves as a primary component of the extracellular matrix, playing crucial roles in maintaining tissue and organ integrity, mediating signal transduction, regulating cell behavior, and tissue repair and regeneration (An et al., 2016). In recent years, natural collagen has found widespread applications in the biomedical field. However, collagen derived from animal sources presents potential risks such as disease transmission and immunogenicity. Moreover, recombinant collagen produced using current technologies often lacks hydroxylation of proline residues and faces challenges in achieving ordered spatial arrangement at the nanoscale (Zhao et al., 2021). Designing collagen peptides at the molecular level offers controllable structures, ease of chemical modification, and excellent biocompatibility. Compared with natural collagen, the chemically synthesized collagen demonstrates superior advantages in terms of structural controllability, batch production feasibility, standardized synthesis processes, and low immunogenicity. Importantly, our synthesized collagen exhibits enhanced thermal stability and structural integrity compared to natural bovine type I collagen. Therefore, the development of self-assembling peptides as biomimetic collagen has been a focal point in tissue engineering research. Among them, homotrimeric collagen peptides are relatively straightforward to design, chemically modifiable, and can form stable triple helical structures, making them widely applicable in hemostatic materials, drug delivery, and biocellular scaffolds (Przybyla et al., 2013; Cejas et al., 2007).

Proliferating cell nuclear antigen (PCNA), initially discovered in the serum of patients with systemic lupus erythematosus, has been found to exist as a homotrimer in various eukaryotic cells, forming a homotrimeric ring structure (Strzalka and Ziemienowicz, 2011). Among the 29 known human collagen types, 21 belong to homotrimeric collagen, with type II collagen being a typical example. Type II collagen is a crucial component of tissues such as cartilage and joints (Sorushanova et al., 2019). Mutations in genes such as COL11A1, COL11A2, and COL2A1 lead to structural defects or insufficient expression of type II collagen, disrupting normal development of bones and connective tissues, leading to type II collagenopathies, with common manifestations including cartilage dysplasia, spinal deformities, short stature, and Stickler syndrome (Acke et al., 2012).

Solid-phase peptide synthesis (SPPS) is a commonly used method for chemical synthesis of peptides, allowing the synthesis of various peptide sequences, including complex or cyclic peptide products (Isidro-Llobet et al., 2019). It finds wide applications in the biomedical field. However, the excessive use of Fmoc-protected amino acids and coupling reagents in SPPS leads to environmental pollution. Additionally, classic organic synthesis methods often involve the use of large quantities of environmentally hazardous materials (Rasmussen, 2018). In peptide synthesis, amino acid coupling can be achieved using acid (e.g., HOBT-DIC reaction system) or base (e.g., DIPEA-HATU reaction system) methods. The HOBT-DIC reaction system, requiring only 45 min to 1 h for complete reaction, offers higher efficiency and resin binding compared to the DIPEA-HATU reaction system, which requires two feedings of 30 min each and is more costly and cumbersome (Martin et al., 2020). Furthermore, carboxyl activation is mainly employed in peptide synthesis to complete the peptide coupling reaction. EDCHCl, due to its water solubility, is widely used in peptide and protein conjugation reactions, showing more success in peptide synthesis. In our study, we synthesized collagen peptides using the EDCHCl/HOBt chemical method, which offers good environmental friendliness and synthesis efficiency.

Research has shown that the design and assembly of homotrimeric collagen peptides often rely on sequences rich in proline and glycine, with (POG)n (PPG)n being the main motif, and insertion of amino acids with specific functional groups such as Cys, Asp, Lys, Phe, and His. These peptides spontaneously or controllably self-assemble into structurally diverse assemblies through interactions such as amphiphilicity, disulfide bonds, electrostatic attraction, π-π stacking, and metal coordination, leading to the formation of complex and variable nanomaterials (Meister et al., 2008; Zykwinska et al., 2014; Zykova et al., 2022). Luo et al. designed amphiphilic collagen peptides containing integrin-binding sequences, which self-assembled into nanofibers promoting cell adhesion (Luo and Tong, 2011). He et al. constructed lanthanide-ion-mediated self-assembling collagen peptides, which formed helical nanorope structures and exhibited periodic arrangement similar to natural collagen, showing great potential in cell imaging, medical diagnosis, and tissue culture (He et al., 2016). Rele et al. designed collagen peptide CPII, which self-assembled into fibrous structures with periodic stripes through electrostatic interactions. O'Leary et al. redesigned the peptide sequence of CPII to obtain peptide KOD, which mimicked all steps of collagen fibrillogenesis, from triple helix to nanofiber to hydrogel (Rele et al., 2007; O'Leary et al., 2011).

Due to advantages such as controllable structure, ease of modification, and absence of viral infection risks, biomimetic collagen materials are increasingly recognized for clinical applications. Kumar et al. designed and self-assembled a collagen peptide that mimicked the extracellular matrix, exhibiting similar effects to animal-derived collagen in promoting platelet adhesion and blood clotting after tissue injury repair (Kumar et al., 2014). Besides promoting tissue regeneration akin to natural collagen, modified biomimetic collagen peptides can be used to encapsulate and transport biomolecules, ensuring the activity of sensitive biomolecules while controlling their release and targeted delivery. Cejas et al. reported collagen peptides Hbyp3 and Hbyp3-11, which self-assembled into nanoscale disc-like structures and formed microcage structures through the addition of Fe(II) ions, allowing control of the release rate of active molecules by modulating the stability of the triple helical structure (Cejas et al., 2007). Pries et al. designed metal-triggered self-assembling collagen peptides as three-dimensional scaffolds for cell culture, enabling cells to maintain good activity and proliferation within the scaffold, with mild EDTA treatment releasing viable cells that retained good activity and proliferation (Pires et al., 2009). The collagen peptides we constructed consist entirely of natural amino acids, eliminating potential toxicity associated with introducing metal ions, making them widely applicable and providing a new strategy for better mimicking the structure and function of collagen in tissue engineering, medical devices, and other fields. Compared to existing collagen mimics or recombinant collagens, our synthesized homotrimeric collagen peptides exhibit unique advantages. While recombinant collagen often lacks proline hydroxylation and struggles with nanoscale structural organization, our peptide design ensures a stable triple helical conformation with excellent thermal stability. Additionally, many biomimetic collagen peptides require metal ion coordination or other modifications to achieve functional self-assembly, which may introduce potential cytotoxicity. In contrast, our peptide consists solely of natural amino acids, reducing biocompatibility concerns and broadening its clinical applicability. These advantages position our material as a promising next-generation biomimetic collagen for tissue engineering, medical devices, and targeted drug delivery.

Circular dichroism (CD) spectroscopy is one of the primary methods for analyzing protein secondary structure and has been widely used in studies on protein conformational changes, protein folding, and enzyme kinetics (Greenfield, 2006). Different secondary structures of proteins exhibit distinct CD spectra. Proteins rich in α-helical structures show negative peaks at 222 nm and 208 nm and a positive peak at 193 nm, while those abundant in antiparallel β-sheet structures exhibit a negative peak at 218 nm and a positive peak at 195 nm (Segatta et al., 2021; Unneberg et al., 2001). For collagen triple helical structures, characteristic positive peaks are observed at 220 nm, with negative peaks at 195 nm. In addition to determining protein secondary structure, CD spectroscopy can be used to determine the stability of protein triple helical structures by tracking the intensity of peaks at 225 nm in real-time as collagen peptide samples are heated. The thermal transition temperature (Tm) reflects the stability of the triple helical structure, with higher Tm indicating better thermal stability (Wachamo et al., 2021). Through analysis of CD spectra at different temperatures, we found that artificially synthesized collagen peptides exhibit stable triple helical structures with good thermal stability, making them excellent biomimetic collagen materials.

The promotion of collagen and elastin synthesis observed in our study suggests that the designed collagen peptide may actively engage cellular signaling pathways involved in extracellular matrix remodeling. The TGF-β/SMAD and MAPK/ERK pathways are well-documented regulators of fibroblast function and collagen deposition (Verrecchia and Mauviel, 2004; Zhou et al., 2024), and they may be involved in mediating the observed effects (Wang et al., 2016). Additionally, synthetic collagen peptides may enhance collagen synthesis by inhibiting collagen-degrading enzymes such as MMPs (Mendes Soares et al., 2022), regulating post-translational modification pathways, and stimulating collagen-synthesizing enzymes. Growth factors like IGF-1 (Miescher et al., 2023) and FGF (Hillege et al., 2020) could further promote fibroblast proliferation and collagen accumulation through synergistic mechanisms. Integrins, particularly α2β1 and αvβ3, are key receptors for collagen peptides, facilitating cell adhesion and activating downstream signaling cascades such as FAK/PI3K/AKT, which regulate ECM homeostasis and fibroblast activity (Chastney et al., 2021). While our study focuses on the structural and functional properties of the synthesized peptide, further research is warranted to elucidate its precise molecular mechanisms in promoting collagen and elastin synthesis.

## 5 Conclusion

The design and synthesis of biomimetic collagen proteins are largely driven by the molecular structure and stability of the triple helix. We successfully designed and synthesized a homotrimeric collagen peptide with stable triple helical structure and good thermal stability. *In vitro* and *in vivo* experiments have confirmed its safety and efficacy. The synthesized peptide’s thermal resilience (Tm = 326.15°C) surpasses natural collagen (Tm = 40.24°C), positioning it as a groundbreaking candidate for scenarios demanding heat resistance, such as surgical implants or sterilizable wound dressings. It represents a high-quality biomimetic collagen material worthy of widespread application in the biomedical field.

## Data Availability

The original contributions presented in the study are included in the article/Supplementary Material, further inquiries can be directed to the corresponding authors.
