# Supplementary material for: Structural and functional analysis of a homotrimeric collagen peptide
Source: Front Bioeng Biotechnol. 2025 Apr 28;13:1575341. doi: 10.3389/fbioe.2025.1575341 (PMC12066645; doi:10.3389/fbioe.2025.1575341)
Supplement: Supplementary file 1 [file Table1.docx]

Supplementary Table 1: Scan Parameters

| Item | Value |
| --- | --- |
| Bandwidth | 1.0nm |
| Measurement Range | Far-UV region scan: 190-260nm |
| Time per Point | 0.5s |
| Accumulation | Sample: 3, PB buffer: 2 |
| Cell Length | Far-UV: 10mm |
| Temperature | Room Temperature |

Supplementary Table 2: SCD Spectra Analysis:

|  | Alpha Helix | Antiparallel Beta Fold | Parallel Beta Fold | Turns | Irregular Coils |
| --- | --- | --- | --- | --- | --- |
| Bovine Type I Collagen | 3.2% | 10.2% | 1.7% | 30% | 52.4% |
| Collagen Peptide | 3.2% | 10.2% | 1.8% | 31% | 52.4% |

Supplementary Table 3: Infrared Spectra Peak Characteristics:

|  | Amide A/ cm-1 | Amide B/ cm-1 | Amide I/cm-1 | Amide II/cm-1 | Amide III/cm-1 |
| --- | --- | --- | --- | --- | --- |
| Bovine Type I Collagen | 3301.22 | 2937.61 | 1633.68 | 1547.54 | 1237.82 |
| Collagen Peptide | 3329.71 | 2949.71 | 1626.5 | 1552.29 | 1231.74 |

Peak Frequency Notes:

Amide A/ cm-1: Absorption peak for OH and NH stretching vibrations.

Amide B/ cm-1: Absorption peak for saturated -C-H stretching vibrations.

Amide I/cm-1: Absorption peak for C=O stretching vibrations on the amide.

Amide II/cm-1: Absorption peak for N-H bending vibrations on the amide.

Amide III/cm-1: Absorption peak for C-O stretching vibrations.
