# Supplementary figures and images for: Structural and functional analysis of a homotrimeric collagen peptide
Source: Front Bioeng Biotechnol. 2025 Apr 28;13:1575341. doi: 10.3389/fbioe.2025.1575341 (PMC12066645; doi:10.3389/fbioe.2025.1575341)

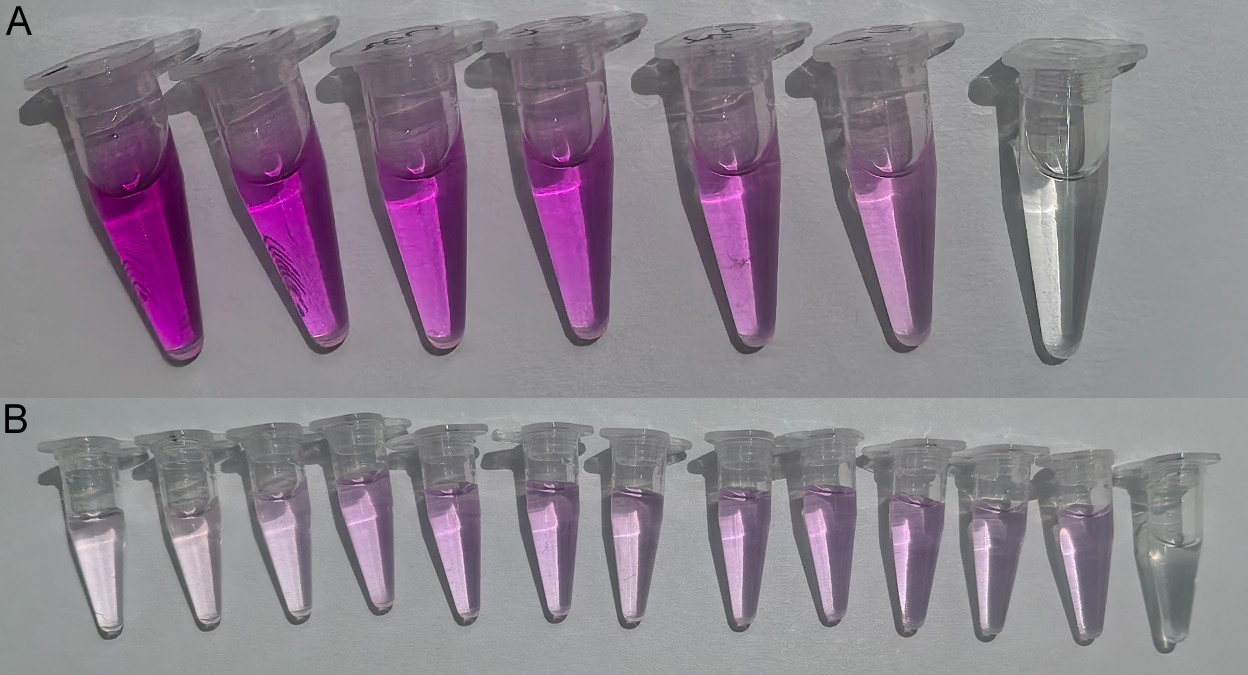

Supplement: Supplementary file 2 [file Image1.jpeg]
